# Experimental Evidence for Seed Metabolic Allometry in Barrel Medic (*Medicago truncatula* Gaertn.)

**DOI:** 10.3390/ijms23158484

**Published:** 2022-07-30

**Authors:** Jean-Baptiste Domergue, Julie Lalande, Daniel Beucher, Pascale Satour, Cyril Abadie, Anis M. Limami, Guillaume Tcherkez

**Affiliations:** 1 Institut de Recherche en Horticulture et Semences, Université d’Angers, INRAe, Institut Agro, 42 Rue Georges Morel, 49070 Beaucouze, France; jean-baptiste@domergue.com (J.-B.D.); julie.lalande@inrae.fr (J.L.); daniel.beucher@agrocampus-ouest.fr (D.B.); pascale.satour@univ-angers.fr (P.S.); cyril.abadie@inrae.fr (C.A.); anis.limami@univ-angers.fr (A.M.L.); 2 Research School of Biology, ANU College of Sciences, Australian National University, Canberra, ACT 2601, Australia

**Keywords:** seed, metabolomics, isotope, elemental composition, size, fluxes

## Abstract

Seed size is often considered to be an important trait for seed quality, i.e., vigour and germination performance. It is believed that seed size reflects the quantity of reserve material and thus the C and N sources available for post-germinative processes. However, mechanisms linking seed size and quality are poorly documented. In particular, specific metabolic changes when seed size varies are not well-known. To gain insight into this aspect, we examined seed size and composition across different accessions of barrel medic (*Medicago truncatula* Gaertn.) from the genetic core collection. We conducted multi-elemental analyses and isotope measurements, as well as exact mass GC–MS metabolomics. There was a systematic increase in N content (+0.17% N mg^−1^) and a decrease in H content (–0.14% H mg^−1^) with seed size, reflecting lower lipid and higher S-poor protein quantity. There was also a decrease in ^2^H natural abundance (δ^2^H), due to the lower prevalence of ^2^H-enriched lipid hydrogen atoms that underwent isotopic exchange with water during seed development. Metabolomics showed that seed size correlates with free amino acid and hexoses content, and anticorrelates with amino acid degradation products, disaccharides, malic acid and free fatty acids. All accessions followed the same trend, with insignificant differences in metabolic properties between them. Our results show that there is no general, proportional increase in metabolite pools with seed size. Seed size appears to be determined by metabolic balance (between sugar and amino acid degradation vs. utilisation for storage), which is in turn likely determined by phloem source metabolite delivery during seed development.

## 1. Introduction

Intense efforts are currently being devoted to improving seed quality and thereby finding traits that best correlate to vigour, germination performance or seedling establishment probability. Amongst morphological traits that have some potential to reflect seed quality is seed size or weight [1]. In fact, some (negative or positive) correlation could be anticipated between seed size and resistance to ageing, viability, vigour or germination performance. Pioneering experiments with barley have shown a link between seed size and vigour [2]. Nevertheless, there seems to be no general rule, since such a correlation depends on species (see examples in wheat, oil palm and maize just below in this *Introduction*). At first glance, larger seeds may reflect larger content in reserve material, such as proteins, starch or lipids, and this trait might thus be seen as beneficial to sustain growth and metabolism due to higher amount of available source carbon (C), nitrogen (N) and sulphur (S). For example, in wheat, larger grain protein and N content has been suggested to correlate to seed vigour [3]. In oil palm, larger seeds are associated with higher germination rate [4]. However, large seed size can also be detrimental, in particular in cereals. In maize, a genetic analysis of germination in relation to kernel size (thousand kernel weight) showed that smaller kernels had higher germination efficiency [5]. In wheat, large grain size correlates with occurrence of a thick starch layer in the endosperm, forming a physical barrier for radicle piercing and thus for germination [6]. In addition, no relationship has been found between grain weight and protein relative content either across varieties or growth conditions, due to the balance between grain number and weight [7].

The complicated, species-dependent relationship between seed weight and performance is potentially problematic for agriculture, since it participates in determining sowing density, which is often monitored in kg m^−2^. Additionally, possible relationships between seed weight and N content are of importance for intercropping, where species with different N remobilisation capabilities are used to improve overall N use efficiency of agrosystems [8]. In that regard, Legumes are important species since in principle, they do not require N fertilisation for germination and seedling establishment, and then provide N to the cereal species later on during the growth cycle, via symbiotic N_2_ fixation [9,10].

In Legumes, a positive correlation has been found between seed weight and seed vigour, germination performance and seedling heterotrophic growth [11,12,13,14,15]. In addition, genome-wide association studies (GWAS) carried out with barrel medic accessions (from the core collection) have suggested a link between seed size, N content and seedling establishment [15,16]. Additionally, a recent GWAS analysis with barrel medic accessions has suggested the involvement of a U12-type spliceosome component in determining seed size, showing a link with mRNA synthesis and maturation [17]. While the link between seed size, cotyledonary reserves and carbon (and nitrogen) provision via remobilisation can be hypothesized for post-germinative events, it is less so for germination stricto sensu since in Legumes, it relies primarily on free amino acids and then soluble sugars [18]. It is thus likely that changes in seed weight are associated with cellular and/or biochemical modifications exerting a control on vigour and/or germinative properties. Seed quality biomarkers from primary C, N and S metabolism have been reviewed to list metabolic pathway that are critical for seed vigour and germination and in Legumes, sugar species seem to be of importance [19]. In a comparison of genetic lines (accessions), metabolites of the raffinose family oligosaccharides (RFO) group have been found to be associated with seed vigour in *M. truncatula* [20]. However, there remains a gap of knowledge as to whether seed weight translates into specific seed metabolome, which can in turn modulate seed quality via metabolic or signalling effects. That is, do metabolic pools scale proportionally to seed weight (and thus keep constant when expressed on a weight basis) or, conversely, is there a metabolic allometry whereby metabolic pools change dramatically when seed weight increases or decreases?

To answer this question, we carried out extensive analyses of elemental composition (C, N, S and H), isotope natural abundance determination (δ^13^C, δ^15^N, δ^34^S, δ^2^H) and exact mass GC-MS based metabolomics on eight accessions of *M. truncatula* (Table 1) and looked at relationships between seed weight and metabolic properties. Our working hypothesis is that seeds of different weight have contrasted biochemical composition, and this must be visible in whole seed metabolome. Additionally, natural isotope abundance is of particular interest to detect changes in metabolic pathways and branching points, without the need for labelling. Typically, δ^13^C reflects water use efficiency, while δ^15^N gives an estimate of N derived from symbiotic fixation as well as fractionations in N metabolism. δ^34^S is a natural tracer of sulphur assimilation from sulphate and S allocation. δ^2^H is a marker of isotopic exchange with water and metabolic fractionations in enzymatic H-transfers. Using modelling and multivariate statistics on the dataset (made of more than 40,000 metabolic individual data), we find that seed metabolic composition is strongly impacted by seed weight, mostly independently of the genetic background.

## 2. Results

### 2.1. Seed Elemental Composition

**Figure 1:**
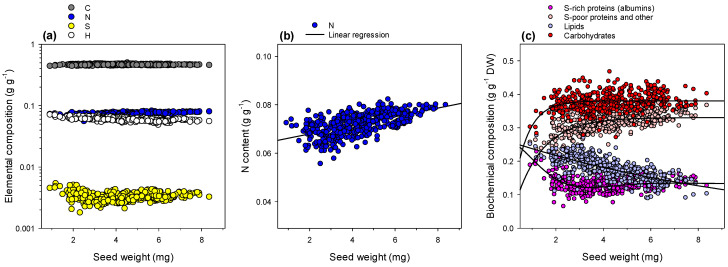
Seed composition. Elemental composition (**a**) measured by elemental analysis, in carbon, nitrogen, sulphur and hydrogen, in mass fraction (note the logarithmic scale to make the four elements of interest more easily visible). (**b**) Magnification of the relationship between N content and seed weight. The black line stands for a linear regression (*R*^2^ = 0.36, *p* < 0.001). (**c**) Biochemical composition in proteins, lipids and carbohydrates calculated from elemental composition. Thick black lines stand for exponential trends, exponential decay or raise to maximum (in all cases, *R*^2^ > 0.96, *p* < 0.0001). In (**c**), major compounds considered are S-rich proteins (albumin type), other nitrogenous compounds (including S-poor proteins, such as vicilins and legumins), total lipids and carbohydrates (both structural and non-structural). Elemental data are plotted against accessions in Supplementary Figure S1.

Elemental composition measured using an elemental analyser either in combustion (C, N, S) or pyrolysis mode (H) is shown in Figure 1, with all accessions plotted together. Individual contents in accessions are shown in Supplementary Figure S1. There was a slight increase in N content and a clear decrease in H content as seed weight increased (Figure 1a), while C remained rather constant, and S was more abundant in very small seeds (smaller than 2 mg). Although slight, the increase in N content with seed weight was significant, starting near 6% in small seeds to 8% in large seeds (i.e., 8 mg) (Figure 1b). We took advantage of elemental composition to convert results into content in major components of known chemical formula (albumin, other proteins + nitrogenous compounds, lipids, carbohydrates), using numerical resolution (Figure 1c). While carbohydrates and albumins always represented about 37% and 13% of seed weight, respectively (except in very small seeds), there was a very clear and progressive increase in S-poor proteins + other nitrogenous compounds (vicilins, legumins, etc.) and a decrease in lipid content as seed weight increased. Overall, there was little difference between accessions (Supplementary Figure S1i). In effect, seed weight was rather similar (no significant differences between accessions), although accession 368 produced the smallest seeds, and accession 544 produced the largest seeds in our dataset. Accessions 736 and 738 had more and less carbon, respectively, and accession 738 had more sulphur (Supplementary Figure S1a–d). As a result, computed biochemical composition suggested more albumins in accession 738.

### 2.2. Isotope Composition

**Figure 2:**
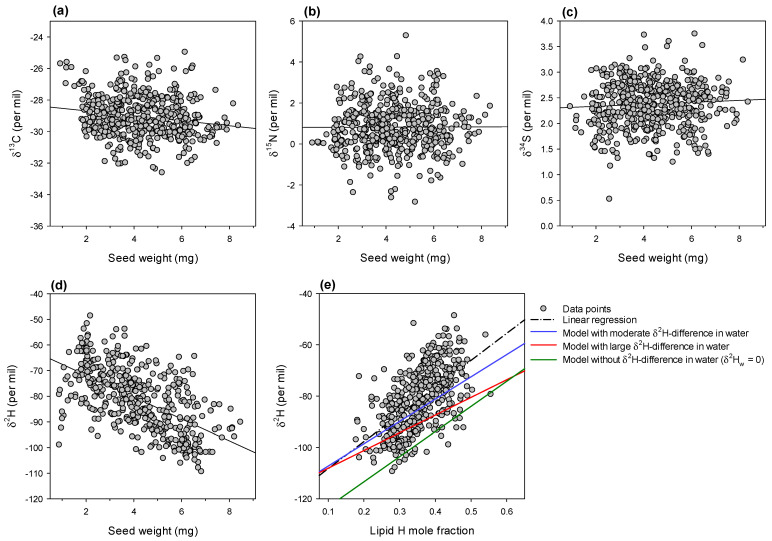
Seed isotope composition (previous page). Natural abundance in ^13^C (**a**), ^15^N (**b**), ^34^S (**c**) and deuterium (^2^H) (**d**) in seeds, plotted against seed weight. Black lines stand for linear regressions (insignificant for ^13^C, ^15^N and ^34^S; significant for ^2^H, *R*^2^ = 0.33, *p* < 0.0001). (**e**) Relationship between hydrogen isotope composition and hydrogen mole fraction represented by lipids in seeds. The dash-dotted black line stands for the linear regression (*R*^2^ = 0.38, *p* < 0.0001), while the coloured solid lines represent the predicted δ^2^H using isotopic mass–balance models.

Natural isotope abundance was determined with an isotope ratio mass spectrometer and expressed in delta values (Figure 2). There was no significant change in δ^13^C, δ^15^N and δ^34^S with seed weight, with considerable scattering of values (Figure 2a–c). The R^2^ value of the linear regression between δ^13^C and seed weight was only 0.03, suggesting that the variation in water use efficiency (i.e., photosynthetic capacity and stomatal conductance) of plants that generated seeds could only explain 3% of seed weight variance. By contrast, there was a strong and significant decline in δ^2^H such that large seeds were naturally ^2^H-depleted and small seeds were ^2^H-enriched (Figure 2d). Since there was no relationship with δ^13^C, it indicates that δ^2^H was not related to stomatal aperture and transpiration but rather to a metabolic post-photosynthetic effect. To clarify this, we took advantage of the lipid content computed in Figure 1 to calculate the mole fraction of H atoms represented by lipids in seeds. There was a strong and significant relationship between the H mole fraction and δ^2^H (Figure 2e), suggesting that ^2^H natural abundance was linked to the contribution of lipids to seed composition (38% of variance explained). Furthermore, points were extremely close to the expected relationship based on isotope fractionation and H isotopic exchange between metabolic intermediates and water (coloured lines in Figure 2). This strongly suggests that the balance between ^2^H-depleted amino acids and ^2^H-enriched lipid precursors was a major determinant of seed δ^2^H. There were no significant differences in isotope composition between accessions (data not shown).

### 2.3. Seed Metabolome

**Figure 3:**
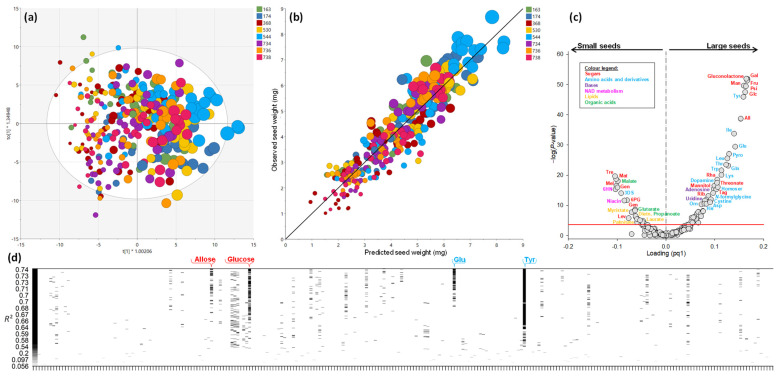
Seed metabolome. (**a**) Score plot of the multivariate analysis (OPLS) showing the discrimination of seed size along axis 1. Seed size is reflected by symbol size, while accessions are shown with colours. The white region stands for Hotelling’s ellipse. (**b**) Relationship between observed seed size and seed size predicted by the statistical OPLS model. The black line stands for the 1:1 relationship. (**c**) Volcano plot showing best discriminant metabolites, with the OPLS loading (*x* axis) and –log(*p*-value) in linear regression (*y* axis). Most important metabolites are annotated, with metabolic families shown in colours. (**d**) R^2^ score plot showing best metabolites in multiple linear regression with variable elimination, implemented in R with the *regsubsets* function. The four best metabolites are annotated on top (allose, glucose, glutamate and tyrosine). Additional information on multivariate analysis is provided in Supplementary Figure S2. In panel (**c**), amino acids and sugars are abbreviated according to the international three-letter code. Other abbreviations are as follows: 3DS, 3-dehydroshikimate; 6PG, 6-phosphogluconate; Homoser, homoserine; Lev, levoglucosan; Orn, ornithine; Pyro, pyroglutamate. Numerical values of loadings and –log(*p*) used in panel (**c**) are tabulated in Supplementary Table S1. In (**a**), note that *x* and *y* axes have been renormalised to allow the visualisation of axes on the scale. A magnified version of this figure is available as Supplementary Figure S2.

The exact mass GC–MS metabolomics of individual seeds was carried out to examine metabolic differences between seeds. All data were expressed relative to seed weight to facilitate result analysis. That is, should a metabolite be in constant amount (i.e., quantity per mg weight) and thus scale proportionally, it should appear as insignificant in subsequent correlation analysis with seed weight. Metabolomics allowed us to identify and quantify 244 analytes (full list in Supplementary Table S1). Multivariate supervised statistics via orthogonal projection on latent structures (OPLS) were performed to look at relationships with seed weight (used as the Y continuous response variable). Despite a few samples standing outside the Hotelling’s ellipse, the OPLS model was highly significant (*P*_CV-ANOVA_ < 10^−20^), explicative (*R*^2^ = 0.85) and predictive (Q^2^ = 0.76), and seed weight was well aligned along axis 1 (Figure 3a). The orthogonal dimension (axis 2) reflected biological variability unrelated to genetics (no discrimination between accessions). Similarly, multivariate non-supervised analysis by principal component analysis (PCA) was also efficient in discriminating seed weight and did not show sample grouping by accession (Supplementary Figure S2a,b).

Additionally, hierarchical clustering conducted using scores of the OPLS model satisfactorily classified samples according to seed weight (Supplementary Figure S2c). The relationship between observed and predicted seed weight was excellent (83% variance explained) (Figure 3b). There was a strong overlapping of accessions in that relationship, although largest and smallest seeds were from accessions 368 and 544, respectively (like in elemental analyses, Supplementary Figure S1i). The best metabolic biomarkers of seed weight were identified using a volcano plot that combines the *p*-value in univariate analysis (linear regression with seed weight) and the loading from the OPLS (Figure 3c). Many metabolites were above the Bonferroni line (red continuous line), above which the false discovery rate is minimised. Large seeds were found to be enriched in hexoses (glucose, galactose, mannose, psicose, fructose, allose), amino acids (many of them) and their derivatives (e.g., dopamine, homoserine, *N*-formylglycine, cystine, ornithine) and nucleosides (adenosine, uridine). Small seeds were enriched in disaccharides (trehalose, maltose, melibiose, β-gentiobiose), lipid precursors (fatty acids: myristate, laurate, palmitate; polar head: diethanolamine), organic acids (including malate) and intermediates of NAD synthesis (niacin, 6-hydroxynicotinate). Multiple linear regression with variable elimination was also conducted and a correlogram is plotted in Figure 3d. Several metabolites appeared to have good R^2^ values (above 0.60), and amongst them, best correlators to seed weight were allose, glucose, glutamate and tyrosine.

### 2.4. Outlook on Amino Acid Metabolism

**Figure 4:**
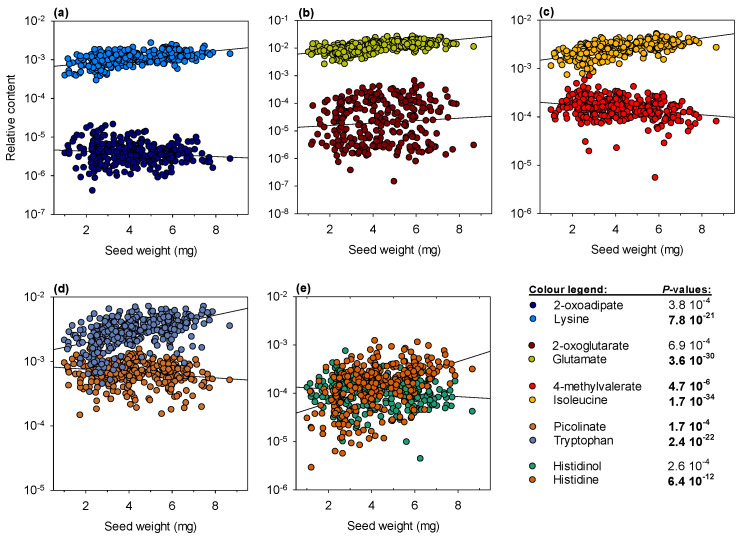
Amino acid degradation products. The relationship between seed weight and relative content in amino acids and their respective degradation products detected in the seed metabolome. Black lines stand for linear regression. Note that a log-scale has been used on the *y*-axis. For each metabolite of interest, *p*-values associated with the regression are indicated on the right-hand side. Values below the Bonferroni-corrected threshold (2.04 × 10^−4^) appear in bold.

To obtain more insight on amino acid metabolism, specific degradation products that could be identified by GC–MS were plotted separately (Figure 4). Selected products include 2-oxoadipate (produced by lysine degradation), 2-oxoglutarate (produced by glutamate transamination), 4-methylvalerate (produced by isoleucine degradation), picolinate (produced by tryptophan degradation) and histidinol (produced by histidine degradation). In all cases, degradation products were more abundant as seed weight decreased, unlike their amino acid counterparts, except for 2-oxoglutarate, which tended to increase slightly. Overall, it indicates a general up-regulation of amino acid catabolism in small seeds.

## 3. Discussion

### 3.1. Seed Weight in Accession Comparisons

Despite having different extreme values in seed weight, the eight accessions examined here did not exhibit significant differences in seed weight overall, as found previously in [16]. Seed weight varied within a 1–9 mg window, which is quite wide. Therefore, when comparing accessions for physiological parameters (e.g., germination assays), it is important to ensure that enough seeds are used to avoid any bias linked to seed weight. It is effectively uncommon to screen seeds for weight or size before carrying out assays (for a recent example of germination assay, see [21]). Since all accessions fell on the same metabolic response curve to seed weight (Figure 3b), selecting seeds of a given weight class (e.g., median 4–5 mg) is probably desirable in physiological assays assessing seed quality. This is even more important for seedling properties, since total available N influences growth rate and emergence [15,16], and we show here that total N does not scale proportionally with seed weight but increases disproportionally (Figure 1b). It is also illustrated in Figure S4, where we conducted multivariate analyses using only seeds with a weight comprised between 3.5 and 5.5 mg. As expected, the discrimination of samples by seed weight is much less efficient, and natural grouping by accessions appears in both unsupervised (PCA) and supervised (OPLS) analyses.

### 3.2. Physiological Consequences of Metabolic Changes with Seed Weight

The most obvious change when seed weight varies is the content in nitrogenous compounds other than albumins (incl. legumins and vicilins) and lipids (Figure 1c). This agrees with the fact that these two components have the same timing of accumulation during *M. truncatula* seed development [22] and thus can be under metabolic competition. Additionally, the previous analysis of seed composition in different lines of *M. truncatula* has suggested a balance between proteins and lipids [23]. This has predictable metabolic consequences during post-germinative reserve remobilisation because the amino acid composition of proteins (albumins, legumins, vicilins) differs, and additionally, lipid remobilisation involves a specific metabolic pathway (β-oxydation and glyoxylic acid cycle). In particular, lipid degradation requires the import of fatty acids into the mitochondrion using carnitines to be broken down, potentially generating more oxidative stress [24,25]. Legumins and vicilins have much more glycine and tryptophan (in % of amino acids in primary sequence), and thus their lower prevalence in small seeds is probably compensated for by de novo synthesis. It is worth noting that small seeds are also depleted in nucleosides (Figure 3c), probably reflecting lower nucleotide synthesis, lower mitotic activity and eventually a smaller number of cells produced during seed development. Accordingly, the contribution of nucleotides as well as nucleic acids is encapsulated in nitrogenous compounds (denoted as ‘S-poor proteins and other’ in Figure 1c) and is higher in large seeds. Cell number counting in the hypocotyl epidermis of embryos has shown that accessions, which can produce very small seeds (368), have a relatively small cell number, in contrast to accessions with the propensity to produce large seeds above 7 mg, such as no. 174 or 738 [26]. Additionally, seed weight and size has been related to mitotic activity, the length of the period of intense cellular division or genes involved in the cell cycle [12,17,27,28]. In the mutant *sbt1.1* of *Medicago truncatula* affected in endosperm subtilase (required to process peptide hormones), seeds were found to be smaller due to a lower cell number [28].

### 3.3. Metabolic Origin of Seed Weight

Seed weight and size is determined by multiple factors, including mating type (self-pollination or outcrossing) [29,30], cell division capability (see above), whole-plant signalling via peptides [31] and key transcription regulators, such as *DASH*, *WRINKLED* or *BIG SEEDS1* [3,12,17,32,33,34]. Interestingly, such regulators are linked to metabolism. For example, *WRINKLED* is not only involved in seed size but also in the balance between proteins and lipid content via a restriction in chloroplastic glycolytic activity (pyruvate kinase) [35]. Seed weight is also related to physiological mechanisms, such as photosynthetic input and photosynthates allocation [36,37], and this should also impact on seed metabolism. Therefore, seed metabolome should not only reflect cellular metabolic programming via genetic regulation but also carbon and nitrogen partitioning via phloem tissues.

**Figure 5:**
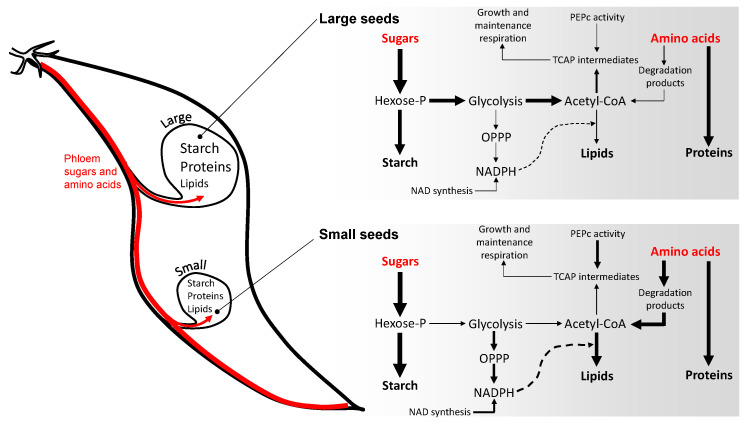
Metabolic changes during maturation associated with seed size. This figure is a simplification based on major products imported from the phloem: sugars and amino acids. Probable relative fluxes are represented with arrow thickness. In small seeds, the lower input from phloem sugars and amino acids (either from sink or source effects) leads to the preferential utilisation of sugars to maintain starch content, and thus an increased utilisation of amino acids as a carbon source, including to synthesise lipids. Increased lipid synthesis goes along a concurrent increase in NADPH production and thus redox power provision. The change in acetyl-CoA partitioning is also associated with an increase in anaplerotic activity, leading to an increased malate content. See main text (*Discussion*) for further details.

Here, the concurrent change in amino acid and sugar metabolism (Figure 3c and Figure 4) strongly suggests a rerouting of amino acid utilisation towards lipid synthesis in small seeds (illustrated in Figure 5). In effect, the relatively modest change in carbohydrates suggests that sugars are preferentially used to store starch at the expense of generation of acetyl-CoA (via glycolysis) during small seed development. This is in agreement with the larger content in sugars that play a role in the control of starch metabolism: in particular, trehalose (Figure 3c) may reflect an increased degradation of trehalose 6-phosphate, an important sugar signal that regulates seed size and the conversion of sucrose into starch [38]. Amino acid degradation then sustains acetyl-CoA synthesis and lipid production. In principle, this should be detrimental to proteins (and thus N content) and lead to a higher carbon cost (CO_2_ production), exaggerating further seed weight limitation. Accrued lipid synthesis requires redox power (NADPH) for fatty acid production, generated from the oxidative pentose phosphate pathway (OPPP), explaining why NAD intermediates are found to be more abundant along with the OPPP intermediate 6-phoshogluconate in small seeds. The intermediates of the OPPP (such as erythrose 4-phosphate) are probably recycled into aromatic compounds, as suggested by 3-dehydroshikimate increase, along with anthranilic acid (*p* = 10^−2.18^) and salicylate (*p* = 10^−4.91^) in small seeds. Additionally, anaplerotic fixation (i.e., PEPC activity producing C_4_ acids to sustain the tricarboxylic acid cycle) has been suggested to be linked to lipid synthesis in Legume seeds [39,40] and accordingly, we find more malate in lipid-rich, small seeds. Taken as a whole, we observe a broad metabolic reorchestration accompanying seed weight, reflecting a compromise to maintain carbohydrates and adjust the protein–lipid balance, a well-known phenomenon found in Legumes under various conditions affecting C/N nutrition [41,42,43,44,45,46,47,48].

This raises the question as to whether such a reorchestration may be caused by a restriction in substrate (sugars, amino acids) provision via phloem sap. Here, the term “provision” encompasses both source (lower delivery by phloem sap) and sink (lower import ability from phloem sap) effects. It has been suggested that in Legumes, the phloem transfer of photosynthates and sugar-cleaving activity (such as invertase) impact on seed size and/or seed set [29,49,50]. In particular, a link has been shown between seed size and invertase and sucrose synthase activities [49,50,51,52,53]. In pea, signalling by trehalose 6-phosphate (via auxin) has been found to control starch production and seed size [38]. In lupine, it has been shown that seed reserve composition is directly affected by the balance between N (asparagine or nitrate) and C (sucrose) supply [46]. Additionally, phloem partitioning to seeds is influenced by both ovule position within the pod and pod position on the mother plant. In fact, variation in seed weight has been observed between pods depending on their position on the plant and their time of development (early or late in the season) [14]. In the genus *Medicago*, there is a peculiar phloem vasculature affecting partitioning to seeds within pods. Like in any Fabaceae, pods are made of two valves and the placental region results from the fusion (dorsal suture) of valves. Thus, along the pod, developing seeds form two imbricated rows, attached to one valve or another (for detailed histology of developing pod in *Medicago truncatula*, see [54]). In *Medicago*, the coiled shape generates high asymmetry between pod valves and seeds attached to the upper valve (i.e., with an even number, when counted from pod peduncle) are larger and have lower propensity to abortion [30].

In both cases (intra-pod and inter-pod seed weight variation), phloem-related mechanisms are involved, and this is reflected by the seed metabolome found here: (i) on the one hand, the general increase in amino acid and sugars in large seeds (Figure 3) pleads in favour of a source effect. (ii) on the other hand, both the absence of sucrose amongst best discriminating metabolites and the change in sugar composition (higher hexoses-to-disaccharide ratio in large seeds) suggest an alteration in sugar interconversions (including by invertase, sucrose synthase or enzymes of RFOs metabolism) and thus in sink strength. Typically, the increase in melibiose in small seeds may suggest that in RFO metabolism, melibiose is cleaved less efficiently, slowing down recycling into glucose and galactose and reflecting lower phloem sucrose (and raffinose) utilisation. Accordingly, HPLC analysis indicates that in small seeds there was an increase in raffinose (6-fold from large to small seeds) and stachyose (up to 2-fold) (Supplementary Figure S5). It should be noted that in Legumes, RFO seems to play an important role. In chickpea, galactinol synthase activity increases during seed development; furthermore, introducing chickpea galactinol synthase in Arabidopsis enhances seed vigour and longevity [55]. Additionally, in *M. truncatula*, using genetic association studies, a correlation has been found between RFO and seed vigour [20]. These observations strongly suggest a beneficial role of galactinol metabolism on seed vigour, likely related to the capability of interconverting sugars, and thus galactinol has been proposed as a biomarker of seed quality [56].

## 4. Material and Methods

### 4.1. Seed Material

Seeds were purchased at the Medicago Biological Resource Centre (INRAe, Montpellier, France). Plants were multiplied to generate seeds in the greenhouse at the plant growth facility INRAe/Institut Agro in Angers (France). Greenhouse air was constantly renewed (with temperature 25/18 °C day/night, and 70% humidity automated control) so that δ^15^N of air N_2_ and δ^13^C in air CO_2_ was kept at 0‰ and ≈ –8‰, respectively. Plants were watered once a day with a nutrient solution (PlantProd^®^ 10-15-30, 1.5 g/L Bioplants, Les Ponts de Cé, France). Three seed sets were used here, cultivated in 2016, 2017, and 2019. There was no significant difference between seed lots in either elemental analyses or isotopic pattern and thus in the paper, seed sets are not distinguished in Figure 1 and Figure 2, and metabolomics were performed on the 2019 set only.

### 4.2. Elemental and Isotopic Analyses

Elemental content and the isotope composition were measured on pre-weighed seeds with a CHONS Elemental Analyzer (EA Vario PYRO cube; Elementar^®^ Elementar, Villeurbanne, France) coupled to an isotope ratio mass spectrometer (IRMS; Isoprime precision, Elementar^®^). Seeds were weighed and folded in tin capsules. The possible offsets of raw IRMS values were corrected using both international IEAE (Vienna) and home-made standards (a sucrose–methionine–glycine mixture, referred to as SMG; chickpea powder; and benzoic acid, both cross-calibrated against caffeine IAEA-600, Ag_2_S IAEA-S-1, benzoic acid IAEA-601 and polyethylene IAEA-CH7). Note that the linearity of IRMS response was checked throughout to be less than 0.02‰ nA^−1^, and furthermore, any possible size effect between samples was very small. All isotopic data were expressed in the conventional delta notation:

δ_sample_ = R_sample_/R_standard_ − 1, expressed in ‰

where R is the heavy/light isotope ratio, and standard material are international reference materials (V-PDB, air N_2_, V-CDT and V-SMOW for ^13^C, ^15^N, ^34^S and ^2^H, respectively).

### 4.3. Metabolomics

Each seed (weighted using a microbalance) was extracted with inox beads in an Eppendorf tube, with methanol:water (80:20, *v*:*v*) with ribitol as an internal standard. After centrifugation, 20 µL of supernatant were poured into a vial (with insert) and spin-dried at 39 °C. Gas chromatography/mass spectrometry (GC–MS) analyses were carried out using a GC–MS-Orbitrap Q Exactive (Thermo Scientific, Waltham, MA, USA). Samples were derivatized (automatically with a preparative robot) with 20 µL of methoxyamine (20 mg mL^−1^ in pyridine; 90 min at 37 °C) and then 30 µL *N*-methyl-*N*(trimethylsilyl)trifluoroacetamide (MSTFA) for 30 min at 37 °C. Prior to injection, 5 µL of alkane mix (14 alkanes from C_9_ to C_36_, 3 µg µL^−1^, Connecticut n-Hydrocarbon Mix, Supelco) were added to each sample to compute the retention index. Analyses were performed by injecting 1 µL in splitless mode at 230 °C (injector temperature) in a TG-5 SILMS column (30 m × 0.25 mm × 0.25 µm; Thermo Scientific) set in a Trace 1310 Series GC (Thermo Scientific). Helium was used as gas carrier with a constant flow of 1 mL min^−1^. After one minute at initial GC oven temperature (70 °C), temperature was raised to 325 °C at 15 °C min^−1^ and finally kept at 325 °C for 4 min. MS analyses were operated in positive polarity in full MS scan mode with the following source settings: mass scan range 50–750 m/z, resolution 60,000, AGC target 1E6, MS transfer line 300 °C and filament delay 4.12 min. Ionisation by electron impact (70 eV) was performed at 250 °C ion source temperature. Metabolites were identified automatically using TraceFinder (Thermo Scientific) using retention time, major characteristic fragment (m/z ion) and a confirmation fragment, with a maximum tolerance of 0.00007 Da. Two (2) quality control (QC) samples (mix of all samples) were injected each 10 samples to check that GC-MS response was constant across sample batches. Additionally, within batches, samples were randomised to avoid any batch-related bias. Metabolomics data were normalised to the internal standard and seed weight.

### 4.4. Calculation of Biochemical Composition

The elemental analysis of seed provided values for %C, %N, %S and %H (in % weight). Observed values were then corrected (divided by 0.85) to account for residual non-extractible seed water (11% weight) and minerals (4%). This was used to back-calculate by mass balance the composition in carbohydrates, proteins and lipids, using the method in [57] used for energetic balance. We used elemental compositions in carbohydrates; S-rich proteins (albumin-like); other nitrogenous compounds, including S-poor proteins; and lipids in weight fractions C_0.4_N_0_S_0_H_0.066_, C_0.505_N_0.187_S_0.027_H_0.069_, C_0.519_N_0.194_S_0.001_H_0.068_ and C_0.760_N_0_S_0_H_0.120_, respectively, corresponding to reference elemental composition of starch, *Medicago* albumin, *Medicago* legumins + vicilins and fatty acids. Calculations were carried out using the Excel solver (non-linear GLC procedure) by the minimisation of the sum of squared differences between modelled and corrected observed composition. The average Euclidian error in fitting was always very small, i.e., 0.03, 0.0005, 10^−6^ and 0.005 for C, N, S and H (values in weight fraction).

### 4.5. Calculation of Predicted δ^2^H

The δ^2^H value was predicted taking into account the δ^2^H of plant water in leaf (δ^2^H_wl_) and pod (δ^2^H_wp_), isotope fractionations in protein and lipid synthesis (Δ) and the proportion of H atoms that could undergo isotopic exchange with water (denoted as *f*), using the same theoretical framework as in [58]. Plant water generally show an isotope enrichment compared to source water because of transpiration (isotope effect in water vaporisation and diffusion). Source metabolites produced by leaves from photosynthetic metabolism inherit the isotope signature of leaf water, corrected for the isotope fractionation in reduction by NADPH, so that:

δ^2^H_source_ = δ^2^H_wl_ · (1 + ε_A_) + ε_A_ (1)

where ε_A_ is the isotope fractionation factor in reduction (–171‰). During the export and utilisation from leaves to pods, there is both exchange with local water and metabolic fractionation, therefore:

δ^2^H_metabolite_ = (1 − *f*) · δ^2^H_source_ + *f* · (δ^2^H_wp_ · (1 + ε_H_) + ε_H_) − Δ (2)

where ε_H_ is the isotope fractionation factor associated with exchange (+158‰). The δ^2^H observed in seeds is the weighted contribution of δ^2^H of proteins, starch and lipids as follows:

δ^2^H_seed_ = (1 − *x*) · δ^2^H_other_ + *x* · δ^2^H_lipids_ (3)

where *x* is the contribution of lipid expressed in H mole fraction (which can be calculated from biochemical composition obtained via elemental analysis). δ^2^H_other_ (average of δ^2^H_protein_ and δ^2^H_starch_) and δ^2^H_lipids_ were calculated using Equation (2) with *f* = 0.33, 0.14 and 0.75 for starch, proteins and lipids, to reflect the fact that (i) one third of H atoms in sugars (OH groups) and (ii) on average two sevenths of H atoms in amino acids and proteins (NH and COOH groups) undergo isotope exchange, and (iii) 75% of H atoms derive from water during trioses conversion to acetyl-CoA and H addition during fatty acid synthesis. The fractionation Δ associated with protein synthesis from amino acids and lipid synthesis from precursors was set at 80‰ and 70‰, respectively [59]. Sugars are used without transformation (other than glycosidic linkage) to synthesise starch, thus we assumed that Δ = 0. The δ^2^H of source rain and tap water was –33‰ (values from NOAA, Nucleus database, average value for Western France). The isotope composition in leaf and pod water was not known. Therefore, in computations, three scenarios were used: (a) moderate leaf–pod water difference (blue line in Figure 2e): leaf water ^2^H-enriched by 60‰ and pod water ^2^H-enriched by 30‰ compared to source water; (b) large leaf–pod water difference (red line in Figure 2e): leaf water ^2^H-enriched by 75‰ and pod water at the same δ^2^H as source water; (c) no leaf–pod difference (green line in Figure 2e): both δ^2^H values were set at 0‰ (33‰ ^2^H-enriched compared to source water). Scenario (a) yielded modelled values that were very close to observed δ^2^H values.

### 4.6. Statistics

Five (metabolomics) to 20 (elemental/isotopic analysis) replicates were done for all accessions and seed weight windows (small, medium, large). Supervised multivariate analysis of metabolomics data was carried out by orthogonal projection on latent structure (OPLS) [60] with Simca (Umetrics), using seed weight as the predicted continuous Y variable and metabolites as predicting X variables. The possible occurrence of statistical outliers was first checked using a principal component analysis (PCA) and look at datapoint outside the 99% confidence Hotelling region. The goodness of the OPLS model was appreciated using the determination coefficient R^2^ and the predictive power was quantified by the cross-validated determination coefficient, Q^2^. The significance of the statistical OPLS model was tested using a χ^2^ comparison with a random model (average ± random error), and the associated *p*-value (*P*_CV-ANOVA_) is reported [61]. Best discriminating features were identified using volcano plots, whereby the logarithm of the *p*-value obtained in univariate analysis (linear regression carried out with R using *glm*) was plotted against the loading (pq1) obtained in the OPLS. In such a representation, best biomarkers have both maximal –log(*p*) and loading values. Most important variables in univariate analysis were identified using multiple linear regression with variable elimination, implemented in R using *regsubsets* (with the following parameterization: force.in = 1, nbest = 100, nvmax = 5, really.big = TRUE).

## 5. Conclusions and Perspectives

Our results show that in barrel medic, seed composition varies allometrically with weight. That is, there are non-proportional changes, not only in protein and lipid content, but also in natural isotope abundance and concentration in many metabolites. This includes sugars species that are believed to be crucial for seed quality. We nevertheless recognise that our analyses used seeds as a whole, while there can be metabolome differences between cotyledons, embryo and seed coat. In particular, seed coat seems to play an important role in seed size in Legumes (reviewed in [62]; see also [63] in Arabidopsis). As such, future studies are warranted to analyse seed parts separately, as has been carried out in other species [64]. Our results also suggest that the seed metabolome reflects metabolic fluxes that prevail during seed development and maturation. In addition to genetics (which have a rather minor influence on seed metabolic content under our conditions), phloem sap movement and utilisation is likely to play an important role in determining seed weight. This aspect warrants further work in the future, such as the metabolomics analysis of phloem sap at different position within pods or at different pod position on the plant stem. Phloem analysis is challenging and other Legumes (such as cowpea) producing larger fruits are amenable to facile phloem exudation via cryopuncture [65]. In terms of isotopic signature, our analysis only used bulk isotope values (entire seeds) but we recognise that it would be interesting to analyse, for instance, amino acids separately (via compound-specific isotope analysis), since their δ^15^N value provides information on metabolic fluxes [66]. This will be addressed in a subsequent study.

## Figures and Tables

**Table 1:** *Medicago truncatula* accessions used in this work. Accession number refers to the reference number at the Medicago Biological Resource Centre (INRAe, Montpellier, France).

Genotype Name	Accession No.	Origin (Location, Altitude, Latitude, Longitude)	Natural Seed Weight Range (mg)
Jemalong A17	738	Australia, NSW	1.1–6.3
DZA045-6	736	Algeria, 100 m, 36.9° N, 7.7° E	1.8–6.4
DZA315-16	734	Algeria, 1070 m, 34.7° N, 0.2° E	1.9–6.1
ESP105-L	544	Spain, 350 m, 38.1° N, 3.8° W	2.3–8.6
F83005-5	530	France, 261 m, 43.6° N, 6.2° E	2.0–6.7
DZA012-J	368	Algeria, 200 m, 36.5° N, 3.2° E	1.0–5.1
SA028064	174	Cyprus, 550 m, 34.8° N, 33.2° E	2.0–7.2
SA022322	163	Syria, 500 m, 35.0° N, 37.1° E	2.0–6.2

## Data Availability

All results are shown in figures and Supplementary Material.

## Supplementary Materials

The following supporting information can be downloaded at: https://www.mdpi.com/article/10.3390/ijms23158484/s1.
