# Supplementary material for: Experimental Evidence for Seed Metabolic Allometry in Barrel Medic (Medicago truncatula Gaertn.)
Source: Int J Mol Sci. 2022 Jul 30;23(15):8484. doi: 10.3390/ijms23158484 (PMC9369157; doi:10.3390/ijms23158484)
Supplement: Supplementary file 1 [file ijms-23-08484-s001.zip › ijms-1804602-supplementary.pdf]

Supplementary material

Table S1. List of analytes detected by exact mass GC-MS in seed metabolome, showing outputs of statistical analyses: pq1, loading of the OPLS analysis associated with the response variable (seed weight); VIP, variable importance for the projection; *P*-value (reg), p-value associated with the linear regression (F-test) with seed weight; –log(*P*), common logarithm of the p-value. A positive (resp. negative) value of pq1 means that the content in the metabolite of interest increases (resp. decreases) with seed weight. D1 and D2 refer to distinct TMS derivatives. TMS, trimethylsilyl.

Analyte name	pq1	VIP	*P*-value (reg)	–log(*P*)
D-Gluconolactone, 4TMS	0.16	2.13	1.17E-52	51.93
D-Fructose, 5TMS D1	0.17	2.15	1.28E-52	51.89
D-Fructose, 5TMS D2	0.17	2.15	2.08E-52	51.68
D-Galactose, 5TMS D1	0.17	2.14	4.62E-52	51.34
D-Glucose, 5TMS D1	0.16	2.08	2.25E-50	49.65
D-Mannose, 5TMS D1	0.16	2.08	2.25E-50	49.65
D-Psicose, 5 TMS	0.16	2.12	3.46E-50	49.46
D-Glucose, 5TMS D2	0.16	2.08	3.08E-48	47.51
L-Tyrosine, 3TMS	0.16	2.04	1.18E-46	45.93
D-Mannose, 5TMS D2	0.15	1.99	1.81E-39	38.74
D-Allose, 5TMS	0.15	1.99	2.03E-39	38.69
L-Isoleucine, 2TMS	0.14	1.86	1.74E-34	33.76
L-Glutamic acid, 3TMS	0.14	1.82	3.61E-30	29.44
L-5-Oxoproline, 2TMS	0.13	1.78	8.89E-28	27.05
L-5-Oxoproline, 3TMS	0.13	1.78	9.33E-28	27.03
L-Leucine, 2TMS	0.13	1.72	2.68E-26	25.57
L-Threonine, 3TMS	0.12	1.69	3.34E-24	23.48
L-Glutamine, 3TMS	0.13	1.74	7.01E-24	23.15
L-Tryptophan, 3TMS	0.11	1.59	2.38E-22	21.62
L-Lysine, 4TMS	0.11	1.60	7.83E-21	20.11
Trehalose, 8TMS	-0.10	1.52	2.03E-20	19.69
Maltose, 8TMS	-0.10	1.50	1.87E-19	18.73
Glucopyranosyl-(1-4 β-D-glucopyranose), 8TMS	-0.10	1.50	1.89E-19	18.72
L-Rhamnose, 4TMS D1	0.11	1.45	2.78E-19	18.56
Malic acid, 3TMS	-0.10	1.34	8.51E-19	18.07
Threonic Acid, TMS	0.10	1.41	3.10E-18	17.51
Erythronic acid, TMS	0.10	1.41	3.10E-18	17.51
Melibiose, 8TMS	-0.10	1.43	2.74E-17	16.56
Dopamine, 4TMS	0.10	1.41	2.97E-17	16.53
Homoserine, 3TMS	0.11	1.39	8.60E-17	16.07
Diethanolamine, 3TMS	0.11	1.39	8.60E-17	16.07
β-Gentiobiose, 8TMS D3	-0.10	1.41	1.09E-16	15.96
D-Mannitol, 6TMS	0.10	1.31	3.00E-16	15.52
Adenosine, 4TMS	0.10	1.38	4.20E-16	15.38
6-Hydroxynicotinic acid, 2TMS	-0.10	1.47	4.66E-16	15.33
6-Phosphogluconic Acid, TMS D2	0.10	1.39	4.81E-16	15.32
D-Tagatose, 5TMS	0.10	1.39	4.81E-16	15.32

N-Formylglycine, 2TMS	0.10	1.28	7.01E-15	14.15
3-Dehydroshikimic acid, 3TMS	-0.09	1.26	8.99E-15	14.05
D-Ribose, 4TMS	0.09	1.18	7.27E-14	13.14
2,3-Diaminopropanoic acid, 3TMS	0.09	1.27	8.88E-13	12.05
L-Cystine, 4TMS	0.09	1.21	9.15E-13	12.04
L-Aspartic acid, 3TMS	0.09	1.25	1.14E-12	11.94
L-Fucose, 4TMS D2	0.08	1.25	1.28E-12	11.89
6-Phosphogluconic Acid, 6TMS D1	-0.08	1.18	1.78E-12	11.75
Niacin, 1TMS	-0.09	1.27	2.29E-12	11.64
L-Ornithine, 3TMS	0.08	1.14	2.58E-12	11.59
L-Histidine, 3TMS	0.08	1.22	6.43E-12	11.19
Uridine, 4TMS	0.09	1.35	9.23E-12	11.03
L-Rhamnose, 4TMS D2	0.08	1.21	2.58E-11	10.59
Salicin, 5TMS	0.08	1.21	2.77E-11	10.56
Citrulline, 3TMS	0.07	1.04	4.41E-11	10.36
β-Gentiobiose, 8TMS D1	-0.06	1.07	2.28E-09	8.64
β-Gentiobiose, 8TMS D2	-0.06	1.07	4.94E-09	8.31
N-Acetyl-L-alanine, 2TMS D1	0.08	1.00	6.30E-09	8.20
N-Acetyl-L-alanine, 2TMS D2	0.08	1.00	6.30E-09	8.20
Pantothenic acid, 3TMS	0.08	1.20	7.82E-09	8.11
Glutaric acid, 2TMS	-0.07	1.21	9.27E-09	8.03
D-Lyxose, 4TMS	0.07	0.94	1.25E-08	7.90
L-Methionine, 2TMS	0.07	0.95	1.50E-08	7.82
Myristic acid, TMS	-0.07	1.16	1.67E-08	7.78
25-Hydroxycholesterol, 2TMS	0.07	1.09	3.55E-08	7.45
D-Xylose, 4TMS D2	0.06	0.92	4.86E-08	7.31
N-Acetyl-D-glucosamine, 4TMS	0.07	1.16	6.12E-08	7.21
Cystathionine, 4TMS	0.07	0.87	1.50E-07	6.82
L-Glycine, 3TMS	0.07	1.04	2.92E-07	6.54
Propanoic acid, TMS D2	-0.06	1.03	3.24E-07	6.49
Xanthosine, TMS	0.07	1.18	3.62E-07	6.44
Diethanolamine, 2TMS	-0.06	1.08	5.74E-07	6.24
N-Acetylaspartic acid, 3TMS	0.06	0.80	5.93E-07	6.23
D-Ribose 5-phosphate, 4TMS D1	0.06	0.91	8.74E-07	6.06
Phosphorylethanolamine, 4TMS	0.06	1.08	9.23E-07	6.03
L-Arginine, 3TMS	0.05	0.93	1.18E-06	5.93
Levoglucosan, 3TMS	-0.08	1.29	1.56E-06	5.81
L-Alanine, 2TMS	0.06	0.98	1.95E-06	5.71
Adenine, 2TMS	0.06	0.85	2.22E-06	5.65
L-Phenylalanine, 2TMS	0.06	1.00	2.36E-06	5.63
4-Methylvaleric acid, TMS	-0.06	0.91	2.72E-06	5.57
Tyramine, 3TMS	-0.06	0.78	3.20E-06	5.50
Lauric acid, 1TMS	-0.06	0.76	6.87E-06	5.16
Uracil, 2TMS	-0.05	1.20	8.15E-06	5.09
4-Aminobenzoic acid, 2TMS	0.06	1.05	8.28E-06	5.08
3-Phosphoglyceraldehyde, TMS D2	0.06	0.78	1.09E-05	4.96

2,4-Dihydroxyacetophenone, 2TMS	-0.06	0.87	1.10E-05	4.96
Salicylic acid, 2TMS	-0.05	0.82	1.22E-05	4.91
Adipic acid, 2TMS	-0.05	0.93	2.07E-05	4.68
L-Serine, 3TMS	0.05	0.80	2.24E-05	4.65
Glycolic acid, 2TMS	-0.05	0.86	2.69E-05	4.57
Thymine, 2TMS	-0.05	1.02	3.15E-05	4.50
Palmitic Acid, 1TMS	-0.06	1.02	3.34E-05	4.48
Heptadecanoic acid, 1TMS	-0.06	0.92	3.93E-05	4.41
Raffinose, 12TMS	0.05	0.91	4.78E-05	4.32
Allantoin, 3TMS	0.07	0.99	5.05E-05	4.30
D-Cellobiose, 8TMS	-0.05	0.80	5.75E-05	4.24
L-Glycine, 2TMS	0.05	0.94	5.95E-05	4.23
Spermidine, 3TMS	-0.05	0.74	1.34E-04	3.87
Picolinic acid, TMS D2	-0.04	1.10	1.74E-04	3.76
p-Coumaric acid, 3TMS	0.05	0.64	2.06E-04	3.69
L-Norleucine, 2TMS	0.04	0.63	2.09E-04	3.68
Urea, 2TMS	-0.04	0.86	2.97E-04	3.53
L-Asparagine, 3TMS	0.05	0.68	3.03E-04	3.52
Glyoxylic acid, 1TMS	-0.04	0.80	3.43E-04	3.46
Isophthalic acid, 2TMS	-0.04	1.01	3.62E-04	3.44
2-Oxoadipic acid, 2TMS	-0.05	0.80	3.81E-04	3.42
L-Cysteine, 3TMS	-0.04	0.88	3.86E-04	3.41
D-Lactose, 8TMS	-0.04	0.70	4.10E-04	3.39
Mevalonolactone, 1TMS	-0.04	0.87	5.00E-04	3.30
2-Oxoglutaric acid, 2TMS	0.04	0.78	6.94E-04	3.16
Ferulic acid, 2TMS	0.05	0.71	7.25E-04	3.14
5-Methylcytosine, 2TMS D2	0.04	0.83	7.51E-04	3.12
Suberic acid, 2TMS	-0.04	1.13	8.77E-04	3.06
Cinnamic acid, 1TMS D1	-0.04	1.07	9.82E-04	3.01
L-Hydroxyproline, 3TMS	-0.04	0.61	1.46E-03	2.83
L-Valine, 2TMS	0.04	0.87	2.07E-03	2.68
Ethanolamine, 3TMS	-0.04	1.09	2.09E-03	2.68
β-Alanine, 3TMS	-0.04	0.62	2.27E-03	2.64
D-Ribulose 1,5-bisphosphate, 4TMS D1	0.03	0.89	2.53E-03	2.60
Histidinol, 2TMS	-0.04	0.87	2.63E-03	2.58
Citraconic acid, 2TMS	-0.03	0.66	3.28E-03	2.48
Erythritol, 4TMS	0.04	0.78	3.74E-03	2.43
Malonic Acid, TMS	0.04	0.61	3.91E-03	2.41
Fumaric acid, 2TMS	-0.02	0.75	4.15E-03	2.38
Succinic acid, 2TMS	0.05	0.87	4.65E-03	2.33
2,4-dihydroxypyrimidine-5-carboxylate, 3TMS	-0.03	0.51	4.65E-03	2.33
L-3-Methoxytyrosine, 3TMS	0.04	0.85	4.68E-03	2.33
Glycerol monostearate, 2TMS	-0.03	0.96	4.77E-03	2.32
Itaconic acid, 2TMS	-0.03	0.52	5.33E-03	2.27
3-Hydroxybenzoic acid, 2TMS	-0.03	1.11	5.51E-03	2.26
Tryptamine, 2TMS	-0.03	0.66	5.52E-03	2.26

D-Arabinose, 4TMS	0.05	0.78	5.81E-03	2.24
Terephthalic acid, 2TMS	-0.03	0.80	6.43E-03	2.19
3-Methylglutaric acid, 2TMS	-0.03	0.45	6.47E-03	2.19
Anthranilic acid, 2TMS	-0.04	0.47	6.59E-03	2.18
Glucosamine, 6TMS	-0.03	0.58	7.06E-03	2.15
Erythrono-1,4-lactone, 2TMS	0.03	0.79	7.72E-03	2.11
D-Glucose-6-phosphate, 5TMS D1	0.03	0.50	8.49E-03	2.07
N-Methylglutamate 3TMS	-0.03	0.50	9.07E-03	2.04
D-Ribose 5-phosphate, 4TMS D2	0.04	0.83	1.09E-02	1.96
Ribulose 5-Phosphate, TMS D2	0.04	0.83	1.09E-02	1.96
1-Monopalmitin, 2TMS	-0.03	0.97	1.15E-02	1.94
Pipecolic acid, 2TMS	-0.03	0.51	1.21E-02	1.92
Glyceraldehyde, 2TMS	-0.04	0.93	1.24E-02	1.91
Tartaric Acid, 4TMS	0.04	0.61	1.28E-02	1.89
6-Phosphogluconic acid, 6TMS D3	0.03	0.54	1.39E-02	1.86
Stearic acid, 1TMS	-0.04	0.56	1.45E-02	1.84
Maleamate, 3TMS	0.03	0.62	1.47E-02	1.83
4-Aminobutanoic acid, 3TMS	0.03	0.63	1.52E-02	1.82
Ascorbic acid, 4TMS D1	0.03	0.58	1.74E-02	1.76
Cytosine, 2TMS	-0.02	1.02	2.00E-02	1.70
Niacinamide, 1TMS	0.03	0.90	2.08E-02	1.68
Pyrazole, 2TMS	-0.03	0.95	2.20E-02	1.66
Methylphosphonic acid, 2TMS	-0.04	0.78	2.52E-02	1.60
Dihydrouracil, 2TMS	0.03	0.55	2.62E-02	1.58
N-Acetyl-L-glutamic acid, 2TMS	0.03	0.46	2.74E-02	1.56
D-Glucuronic acid β-lactone, 3TMS	0.03	0.38	2.84E-02	1.55
Methyl-decanoate	-0.03	0.59	3.24E-02	1.49
Homocysteine, 3TMS	0.03	0.42	4.38E-02	1.36
D-Xylose, 4TMS D1	0.02	0.62	4.57E-02	1.34
Pyrrole-2-carboxylic acid, 2TMS	-0.03	0.92	4.69E-02	1.33
L-Norvaline, 2TMS	-0.03	1.00	5.50E-02	1.26
Isocitric acid, 4TMS	-0.02	0.46	5.69E-02	1.25
Squalene, 1TMS	-0.03	0.79	5.90E-02	1.23
2-oxobutanoate, 2TMS	-0.03	1.00	6.23E-02	1.21
Possible glucosyl-ferulic acid, 3TMS	0.02	0.40	6.36E-02	1.20
Ethylmalonic acid, 2TMS	0.01	0.50	6.42E-02	1.19
α-Tocopherol, 1TMS	0.02	0.43	6.53E-02	1.19
Sorbic acid, 1TMS	-0.01	0.51	6.79E-02	1.17
D-Fructose 6-phosphate, 4TMS	-0.02	0.73	7.02E-02	1.15
Glucosamic acid, 6TMS	-0.02	0.76	7.13E-02	1.15
Glycerol, 3TMS	0.02	0.54	7.50E-02	1.13
Myo-inositol, 6TMS	0.03	0.79	7.87E-02	1.10
Pentanoic acid, 1TMS	-0.03	1.00	8.30E-02	1.08
3-Phosphoglyceraldehyde, 2TMS D1	-0.02	0.81	8.96E-02	1.05
Cinnamic acid, 1TMS D2	-0.03	0.69	9.25E-02	1.03
Taurine, 3TMS	0.02	0.90	9.26E-02	1.03

Cadaverine, 4TMS	-0.02	0.35	1.09E-01	0.96
Inosine, 4TMS	-0.03	0.52	1.14E-01	0.94
L-Cysteine, 2TMS	-0.02	0.99	1.18E-01	0.93
Caffeic acid, 3TMS D1	-0.02	0.94	1.29E-01	0.89
L-Proline, 2TMS	-0.02	0.35	1.30E-01	0.89
Glyceric acid, 3TMS	-0.01	0.92	1.31E-01	0.88
p-Coumaric acid, 2TMS D2	-0.02	0.97	1.36E-01	0.87
Orotic Acid, 3TMS	-0.01	0.27	1.36E-01	0.87
Urocanic Acid, 2TMS	0.01	0.28	1.44E-01	0.84
Mandelic acid, 2TMS	0.00	0.37	1.47E-01	0.83
Hydroxypyruvic acid, 2TMS	0.01	0.52	1.48E-01	0.83
2-Hydroxybutyric acid, 2TMS	-0.01	0.68	1.50E-01	0.82
2,6-Dimethylphenol	-0.02	0.42	1.54E-01	0.81
HMTBA, 2TMS	-0.01	0.17	1.55E-01	0.81
Caffeine	-0.01	0.27	1.62E-01	0.79
Propanoic acid, 1TMS D1	0.00	0.42	1.63E-01	0.79
Pyridoxine, 3TMS	0.02	0.30	1.65E-01	0.78
Arachidic acid, 1TMS	-0.02	0.72	1.86E-01	0.73
Hydrocinnamic acid, 2TMS	-0.02	0.81	1.89E-01	0.72
Pyruvic acid, 2TMS	-0.01	0.35	1.89E-01	0.72
L-Fucose, 4TMS D1	-0.03	0.62	1.89E-01	0.72
2-Methylglutaric acid, 2TMS	-0.01	0.19	2.05E-01	0.69
2,6-Dimethylaniline	-0.01	0.31	2.05E-01	0.69
Glucosamine, 4TMS	0.02	0.27	2.25E-01	0.65
Nonanoic acid, 1TMS	-0.07	1.20	2.92E-01	0.54
Benzoic acid, 1TMS	-0.02	0.79	3.04E-01	0.52
Aconitic acid, 3TMS	-0.01	0.79	3.24E-01	0.49
5-Hydroxylysine, 4TMS	-0.01	0.52	3.32E-01	0.48
Decanoic acid, TMS	-0.02	0.53	3.55E-01	0.45
Sedoheptulose-7-Phosphate, 6TMS	0.01	0.82	3.56E-01	0.45
Salicylamide, 2TMS	-0.01	0.22	3.63E-01	0.44
Sucrose, 8TMS	-0.01	0.77	3.79E-01	0.42
Stigmasterol, 1TMS	-0.02	0.72	3.81E-01	0.42
Sinapinic acid, 2TMS D2	0.01	0.24	3.82E-01	0.42
N-Acetyl-L-phenylalanine, 1TMS	-0.01	0.15	3.90E-01	0.41
Citric acid, 4TMS	-0.01	0.57	3.95E-01	0.40
4-Hydroxyphenylacetic acid, 2TMS	-0.01	1.04	4.11E-01	0.39
Ascorbic Acid, 4TMS D2	-0.02	0.53	4.18E-01	0.38
O-Acetylserine, 2TMS	0.02	0.51	4.29E-01	0.37
Putrescine, 4TMS	-0.01	0.34	4.44E-01	0.35
Deoxycholic acid, 3TMS	-0.01	0.94	4.59E-01	0.34
2-Aminobutanoic acid, 2TMS	-0.01	0.28	4.60E-01	0.34
L-Threitol, 4TMS	-0.01	0.52	5.17E-01	0.29
4-Coumaric acid, 2TMS D1	-0.01	0.33	5.34E-01	0.27
D-Pinitol, 5TMS	0.02	0.51	5.42E-01	0.27
Caffeic acid, 2TMS	-0.01	0.33	5.54E-01	0.26

Galactaric acid, 6TMS	0.01	0.71	5.60E-01	0.25
2,3-Dihydroxybenzoic acid, 3TMS	-0.01	0.40	5.70E-01	0.24
Phtalic-like benzoic acid, 1TMS	-0.02	0.53	5.72E-01	0.24
Purine, 9TMS	0.01	0.13	5.83E-01	0.23
Thiazole, 4-methyl-2-(1-methylethyl)-	-0.01	0.58	6.08E-01	0.22
D-Glucose-6-phosphate, 5TMS D2	0.01	0.86	6.09E-01	0.22
Petroselinic acid, 1TMS	-0.01	0.29	6.42E-01	0.19
Heptanoic acid, 1TMS	-0.04	0.84	6.43E-01	0.19
Ferulic acid, 2TMS	0.00	0.84	6.51E-01	0.19
D-Citramalic acid, 3TMS	0.00	0.46	6.54E-01	0.18
Palmitelaidic acid, 1TMS	0.00	0.27	6.88E-01	0.16
D-Gluconic acid, 6TMS	0.00	0.86	7.42E-01	0.13
Saccharic acid, 6TMS	0.00	0.86	7.43E-01	0.13
Chenodeoxycholic acid, 3TMS	0.01	0.55	7.43E-01	0.13
Dicyclohexylamine	0.00	0.18	7.75E-01	0.11
Octopamine, 4TMS	-0.01	0.09	7.95E-01	0.10
D-Erythrose-4-phosphate, 3TMS	0.00	0.42	8.68E-01	0.06
Quinic acid, 5TMS	0.00	0.25	8.82E-01	0.05
Picolinic acid, 1TMS D1	-0.03	1.01	8.92E-01	0.05
Allocholic acid, 4TMS	-0.01	0.28	9.40E-01	0.03
Hexitol, 6TMS	-0.02	0.62	9.69E-01	0.01
Ornithine lactam, 2TMS	-0.01	0.21	9.69E-01	0.01
Methyl vanillate, 1TMS	0.00	0.23	9.90E-01	0.00

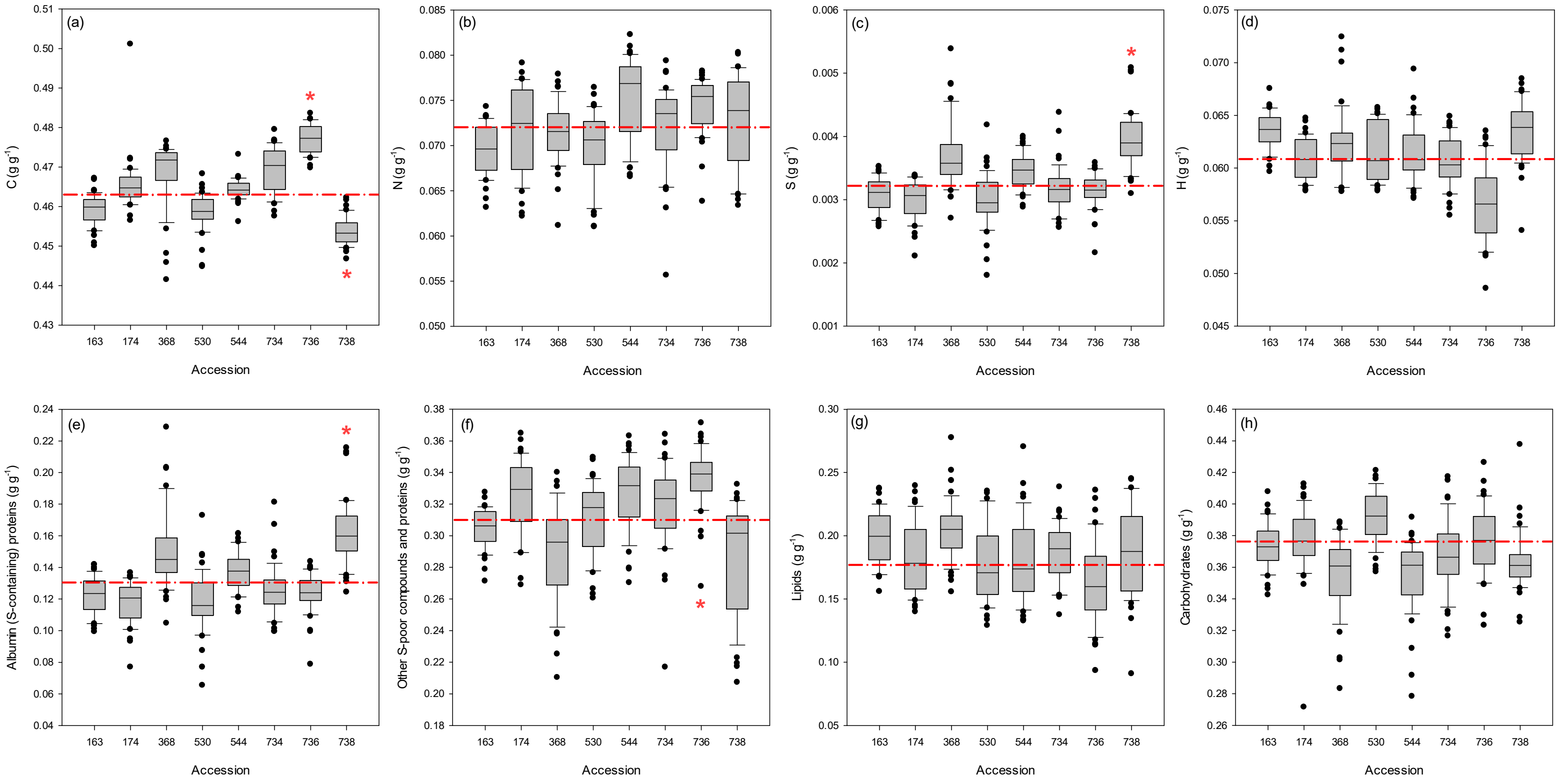

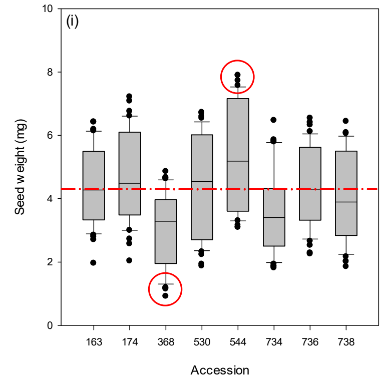

Figure S1. Seed composition and weight shown by accession. C, N, S and H elemental content (in mass fraction, g g^{-1} DW) (a-b), and biochemical composition calculated therefrom (e-h): albumin-like proteins (S-rich) (e), S-poor proteins + other nitrogenous compounds (f), total lipids (g) and total carbohydrates (h). Data are represented as whisker plots (median, first and last quartiles) and the average across accession is shown as a dash-dotted line. Accessions that are significantly different from others are shown with a red asterisk (one-way ANOVA, $P<0.01$). Note the particular composition of seeds from accession no. 738, depleted in C, enriched in S and thus in albumins. Seed weight is also shown in panel (i), in mg. Red circles show minimal (in accession no. 368) and maximal (in accession no. 544) observed values of seed size.

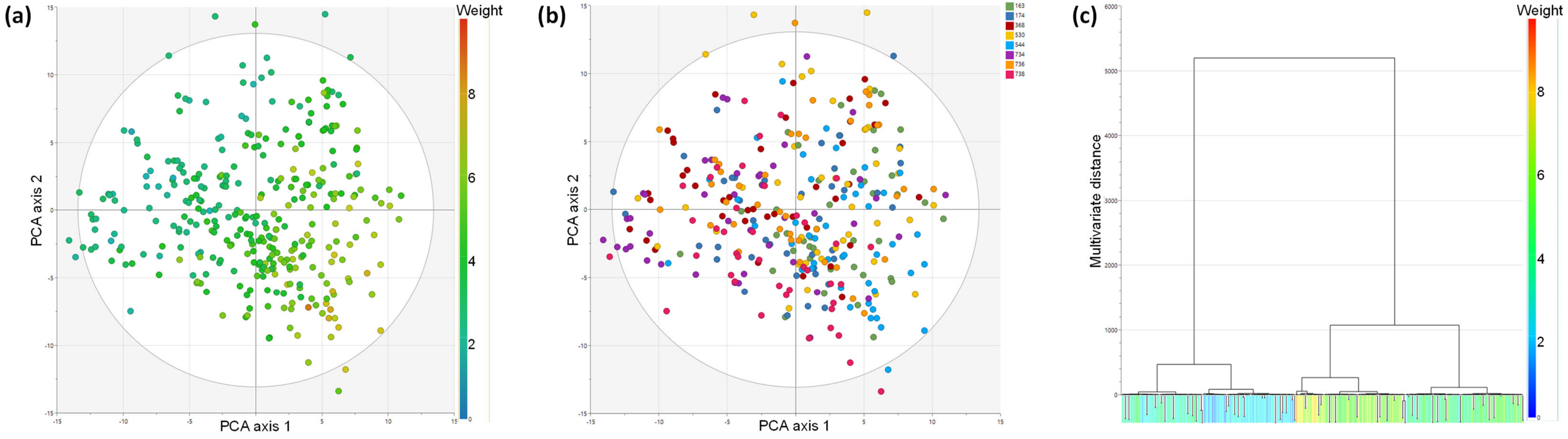

Figure S2. Multivariate analysis and sample clustering. (a) Score plot (axis 1 vs. axis 2) of the principal component analysis (PCA) using seed metabolome, with samples coloured by seed size (colour scale on right from small, blue, to big seeds, red). (b) Same as in (a) but coloured by accessions. Note the absence of accession grouping, with all accessions scattered across the graph. (c) Clustering analysis of samples based on latent variables generated by the OPLS. Samples are coloured according to seed size (colour scale on right) as in (a). Note the very good clustering performance, with clear size groups and little misclassification. In (a) and (b), note that *x* and *y* axes have been renormalised to allow visualisation of axes on the scale.

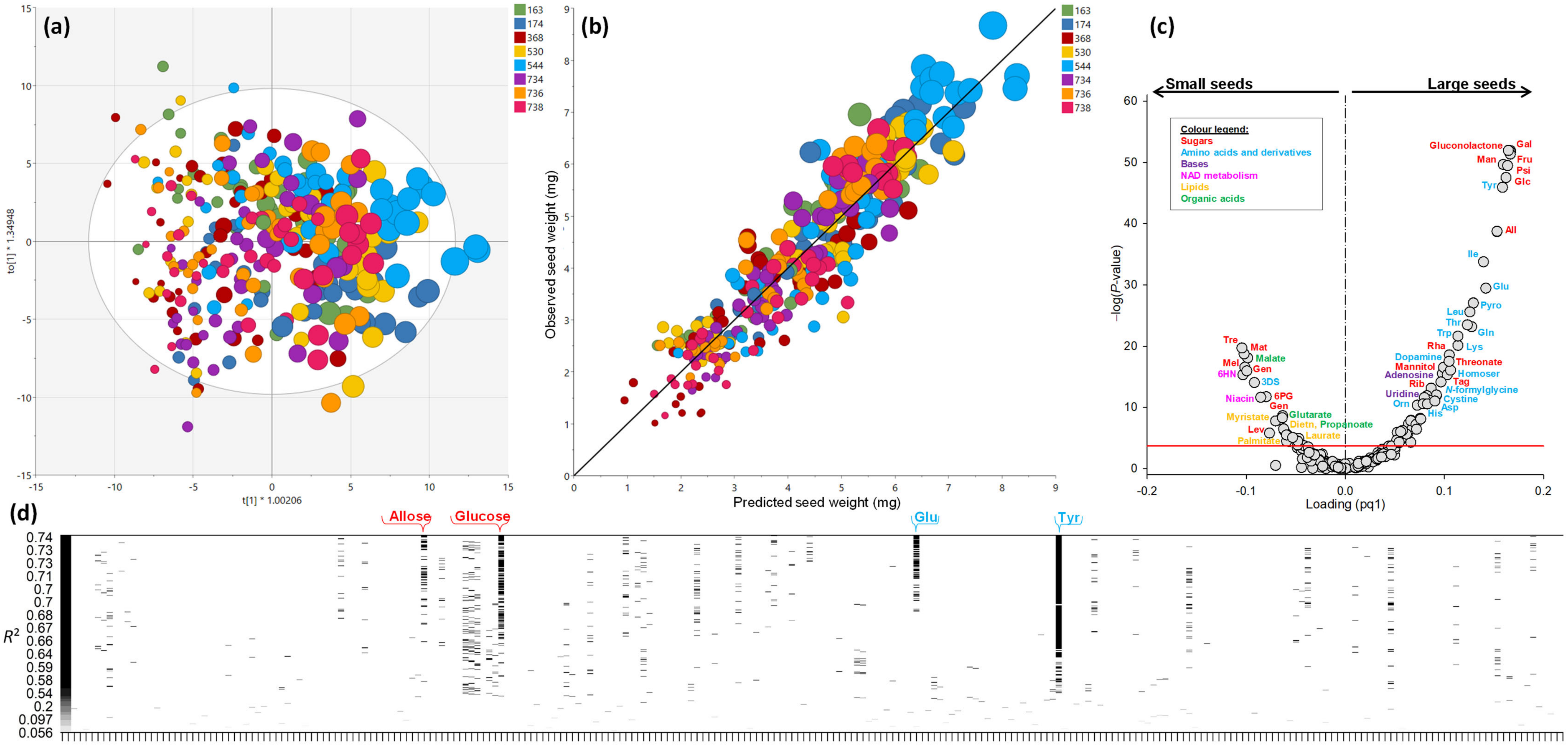

Figure S3. Magnification of Fig. 3 (same legend as Fig. 3).

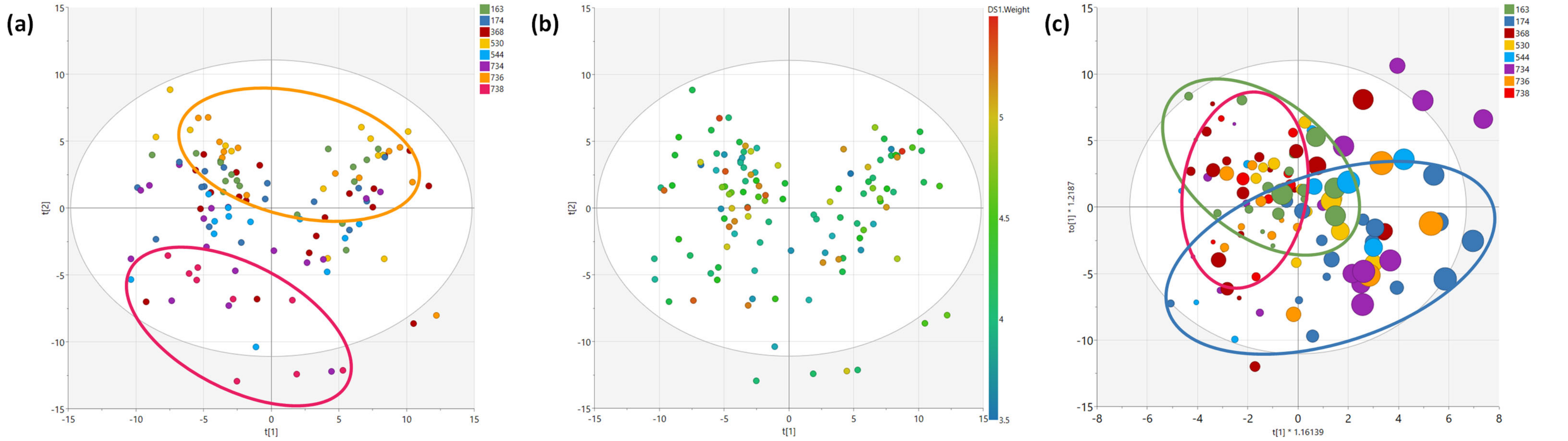

Figure S4. Multivariate analysis of metabolome, using seed on only one weight range (3.5-5.5 mg). (a) Principal component analysis (PCA), showing axes 1 and 2 and partial grouping of samples by accessions (ellipses show examples with accessions 736 and 738). (b) Same PCA as in (a) but colouring by seed weight showing the absence of natural grouping by weight. (c) supervised analysis by OPLS using weight as a Y response variable, showing accession is still a driver of sample discrimination (examples of accessions 163, 738 and 174 shown with ellipses). The performance of the OPLS is $R^2 = 0.65$, $Q^2 = 0.11$ and $P_{CV\text{-}ANOVA} = 0.011$.

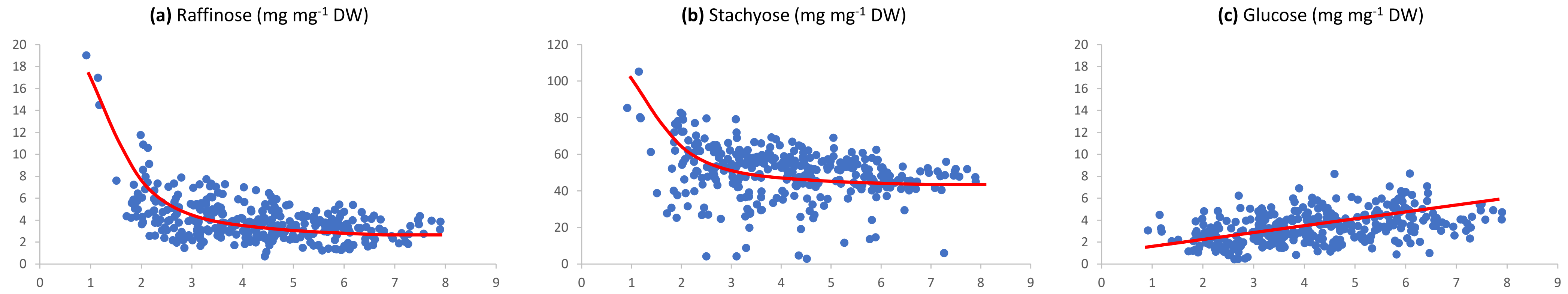

Figure S5. Absolute quantitation of sugars by HPLC: raffinose (a), stachyose (b) and glucose (c) plotted against seed weight, in mg. The red line stands for the general trend (hyperbolic for stachyose and raffinose; linear for glucose). The analytical HPLC method used was the same as in [19].
